# Detection of Oseltamivir-Resistant Pandemic Influenza A(H1N1)pdm2009 in Brazil: Can Community Transmission Be Ruled Out?

**DOI:** 10.1371/journal.pone.0080081

**Published:** 2013-11-11

**Authors:** Thiago Moreno L. Souza, Paola C. Resende, Natalia Fintelman-Rodrigues, Tatiana Schaffer Gregianini, Nilo Ikuta, Sandra Bianchini Fernandes, Ana Luisa Furtado Cury, Maria do Carmo Debur Rosa, Marilda M. Siqueira

**Affiliations:** 1 Laboratório de Vírus Respiratórios e do Sarampo, Instituto Oswaldo Cruz/Fiocruz, Rio de Janeiro, Rio de Janeiro, Brazil; 2 Laboratório Central de Saúde Pública do Estado do Rio de Grande do Sul, Fundação Estadual de Produção e Pesquisa em Saúde Seção de Virologia, Porto Alegre, Rio Grando do Sul, Brazil; 3 Laboratório Central de Saúde Pública do Estado de Santa Catarina, Florianópolis, Santa Catarina, Brazil; 4 Laboratório Central de Saúde Pública do Estado de Minas Gerais, Instituto Octávio Magalhães e Fundação Ezequiel Dias, Belo Horizonte, Minas Gerais, Brazil; 5 Laboratório Central de Saúde Pública do Estado do Paraná, Curitiba, Paraná, Brazil; 6 Universidade Luterana do Brasil, Porto Alegre, Rio Grande do Sul, Brazil; The University of Tokyo, Japan

## Abstract

Although surveillance efforts that monitor the emergence of drug-resistant strains of influenza are critical, systematic analysis is overlooked in most developing countries. We report on the occurrence of strains of pandemic influenza A(H1N1)pdm09 with resistance and decreased susceptibility to oseltamivir (OST) in Brazil in 2009, 2011 and 2012. We found 7 mutant viruses, 2 with the mutation S247N and other 5 with the mutation H275Y. Most of these viruses were from samples concentrated in the southern region of Brazil. Some of these resistant viruses were detected prior to the initiation of OST treatment, suggesting that community transmission of mutant viruses may exist. Moreover, we show that one of these OST-resistant (H275Y) strains of A(H1N1)pdm09 was discovered in the tri-border region between Brazil, Argentina and Paraguay, highlighting that this strain could also be found in other Latin American countries. Our findings reinforce the importance of enhanced antiviral resistance surveillance in Brazil and in other Latin American countries to confirm or rule out the community transmission of OST-resistant strains of A(H1N1)pdm09.

## Introduction

Influenza causes respiratory tract infection and is associated with high rates of morbidity and mortality, which can be more severe in pandemic periods. Although vaccines against influenza are available, changes in their antigenic composition are a necessary consequence of viral escape from immune response – leading to mismatch between the vaccine and circulating strains [1]. Influenza vaccination is generally recommended for individuals at higher risk of influenza-associated complications [2]. Therefore, anti-influenza drugs are essential for prophylaxis and therapeutic interventions [3]. Neuraminidase inhibitors (NAIs), such as oseltamivir (OST), are the main anti-influenza drugs in clinical use [3], as resistance to adamantanes has become common [3]. Before the 2009 pandemic, OST-resistant strains of seasonal human influenza A(H1N1) viruses had already been detected [3]. Since the 2009 pandemic, the use of OST has grown, potentially most importantly imposing selective pressures on the pandemic influenza A(H1N1)pdm09 virus [3]. Therefore, enhanced surveillance capacity to detect the emergence of NAI-resistant strains of A(H1N1)pdm09 should be developed, especially in developing countries, where the scenario is even more challenging due to limited resources and/or laboratory/epidemiological capacities for surveillance. The global circulation of OST-resistant strains of A(H1N1)pdm09 is approximately 1 % [4,5], and concerns about community circulation of these agents have been raised [4,5]. It is unknown for most of the developing countries if there is community circulation of these agents. In 2009 and 2010, our study on immunocompromised, hospitalised or deceased patients did not detect the circulation of OST-resistant strains of A(H1N1)pdm09 in Brazil [6,7]. Nevertheless, our more recent data demonstrated that community transmission of mutant viruses might occur in Brazil. In this article, we discuss possibilities of community transmission in the discovery context of H275Y and S247N viruses. 

## Material and Methods

### Ethics statement

Ethic committee approval and need for informed consent have been waived for this study because influenza surveillance is covered by Brazilian public health policies and all data were analysed in an anonymous fashion. This is in compliance with Decree 05 of February 21, 2006 the Secretariat of Health Surveillance of the ministry of health [8]. This states that: “The results of laboratory tests of disease of immediate notification listed in Annex III of this Decree shall be notified by the national reference laboratories”. Within Annex III, in sub-item II, human influenza is listed as a disease for which of results of samples from research outbreaks should be notified. The mentioned Decree is update from time to time according to needs imposed by public health and preceded by the Brazilian Law No. 6,259, of October 30, 1975 [9]. The second article of this law states that: “the action of epidemiological surveillance comprehends the information, investigations and necessary surveys for planning and evaluation the measures of disease control and situations of health problems”, which is the case when community transmission of OST-resistant strains of influenza seems to be occurring. 

### Patients and data collection

Our laboratory is the National Influenza Center (NIC) in Brazil, we continuously receive a sub-set of samples from the influenza surveillance system from individuals with fever (>37.8°C) and respiratory influenza-like illness [10], who have been treated according to Brazilian guidelines [11]. The samples were accompanied by clinical-epidemiological forms containing at least some basic information, such as patient initials, gender, age, city/state of onset of illness and the dates of the beginning of the symptoms and sample collection. Other clinical characteristics of the patients were collected non-systematically. It is important to note that we used convenience samples for our study, which makes our analysis more convenient for research purposes than as final epidemiological data.

### Sample collection and diagnosis

Nasopharyngeal Dacron swabs or aspirates (NPAs) were collected, and RNA was extracted using a viral RNA mini kit (Qiagen, CA), according to the manufacturer’s instructions. RNA was eluted in 10 mM Tris-HCl, pH 8.0, with 1 mM EDTA (TE buffer) and stored at -70°C. This RNA was used for one-step Real-time RT-PCR assays for influenza subtyping according to the World Health Organisation (WHO) recommendations [10]. 

### Cells and virus isolation

Madin-Darby canine kidney (MDCK) cells were cultured in Dulbecco’s modified Eagle’s medium (DMEM; GIBCO, Grand Island, NY) supplemented with 10 % foetal bovine serum (FBS; Hyclone, Logan, Utah), 100 U/mL penicillin and 100 µg/mL streptomycin and were incubated at 37 °C in 5 % CO_2_ [10]. Virus isolation was performed in either 9-day-old embryonated eggs or in MDCK cells, as previously described [10]. We confirmed viral isolation using haemagglutination, neuraminidase activity or real-time RT-PCR assays [10,12,13]. Viruses were passaged no more than three times.

### Functional antiviral assay

To determine the IC_50_ values of our samples to OST carboxylate, we performed functional antiviral assays using the NA-Star^TM^ assay kit (Life Technologies, CA), according to the manufacturer’s instructions. Briefly, the viral isolates obtained from MDCKs or embryonated eggs were centrifuged at 800 x *g* for 10 min to remove cellular debris. Next, the neuraminidase (NA) activities in these samples were titred as a two-fold dilution to determine the working dilution of the virus. After that, the NA activity of the viral isolates was measured in the presence of different concentrations of OST carboxylate. For comparison, assays with wild-type and resistant standard strains of influenza A(H1N1)pdm09 were run in parallel to validate the IC_50_ values of our isolates, as recommended [10]. The WT (H275) and resistant (H275Y) strains of A(H1N1)pdm09 virus A/Perth/265/2009 and A/Perth/261/2009, respectively, were kindly donated by isirv-AVG (formally the NISN group). 

### Molecular antiviral assays

Single nucleotide polymorphisms (SNP) in the NA gene were analysed by pyrosequencing, as described previously [14]. The RT-PCR was performed using biotinylated primers and SuperScript III/ Platinum Taq DNA Polymerase in a one-step reaction (Invitrogen, CA). The biotinylated RT-PCR products were then mixed with streptavidin and washed to obtain a biotinylated single-stranded DNA. This DNA was used as a template for hybridisation to residue-specific sequencing primers [14].

Alternatively, the whole NA gene was sequenced by Sanger sequencing according to a protocol described elsewhere [15]. The amplified RT-PCR products were purified using the QIAquick PCR Purification kit (QIAGEN, Valencia, CA) and sequenced using a BigDye Terminator v3.1 Cycle Sequencing kit (Life technologies, CA). The products were analysed in an ABI Prism 3130XL genetic analyser (Life technologies). The dataset generated were assembled in Sequencher 5.0 software (GeneCodes Corporation, Michigan, USA) with a NA reference sequence, A/California/4/2009 (GenBank accession number: FJ966084). Of note, N1 numbering was used for NA throughout this study.

## Results

### Sampling and clinical data

From 2011 forward, our laboratory-based influenza antiviral resistance surveillance became broader than in previous years [7] because every current A(H1N1)pdm09-positive NPA received by our laboratory, from patients in 4 out of 5 regions of Brazil (498 cases in total: 163 from 2011 and 335 from 2012), was screened for H275Y and S247N mutations in the neuraminidase (NA) gene. These mutations were chosen for initial investigation because H275Y can lead to resistance to OST [16], and S247N was described early in the pandemic as decreasing viral susceptibility to OST [16,17] and, more recently, as a potentiator of H275Y-imposed resistance against OST [18]. In line with the World Health Organization (WHO), we considered the detection of H275Y and S247N as predictive of strains with resistance and decreased susceptibility to OST, respectively [16,17]. We managed to successfully perform pyrosequencing analysis of 325 and 300 samples to detect the H275Y and S247N mutations, respectively. 

The studied samples belonged to patients with a median age of 20 years old [ranging from 0 to 79 years old (min. and max.) or from 3 to 37 years old (quartile deviation)] – representing the majority of individuals not covered by Brazilian policy to receive influenza vaccination [2]. Approximately 47 % of the individuals were males. The temporal and geographical distribution of our samples was representative of influenza activity in Brazil in 2011 and 2012. Samples collected from May to August (autumn to winter in Brazil) accounted for 70 % of the specimens. Approximately 80 % and 8 % of the samples were from the southern and southeastern regions, respectively (the remaining 12 % were from northern and northeastern regions). Of the patients with a completed standardised data collection (231 A(H1N1)pdm09-confirmed patients), 47 % displayed severe acute respiratory infection (SARI) during the onset of illness, 21 % had been vaccinated against influenza, 20 % were hospitalised, 11 % had other predisposing conditions (HIV/AIDS, cancer, Down syndrome and heavy drug users) and 6.6 % were deceased. Systematic information about the use of OST therapy was rarely collected (5.6 %), which may reflect the absence of this specific field in the standardised data collection form and negligence concerning a critical issue of influenza surveillance. 

### Detection of mutant viruses

**Figure 1:**
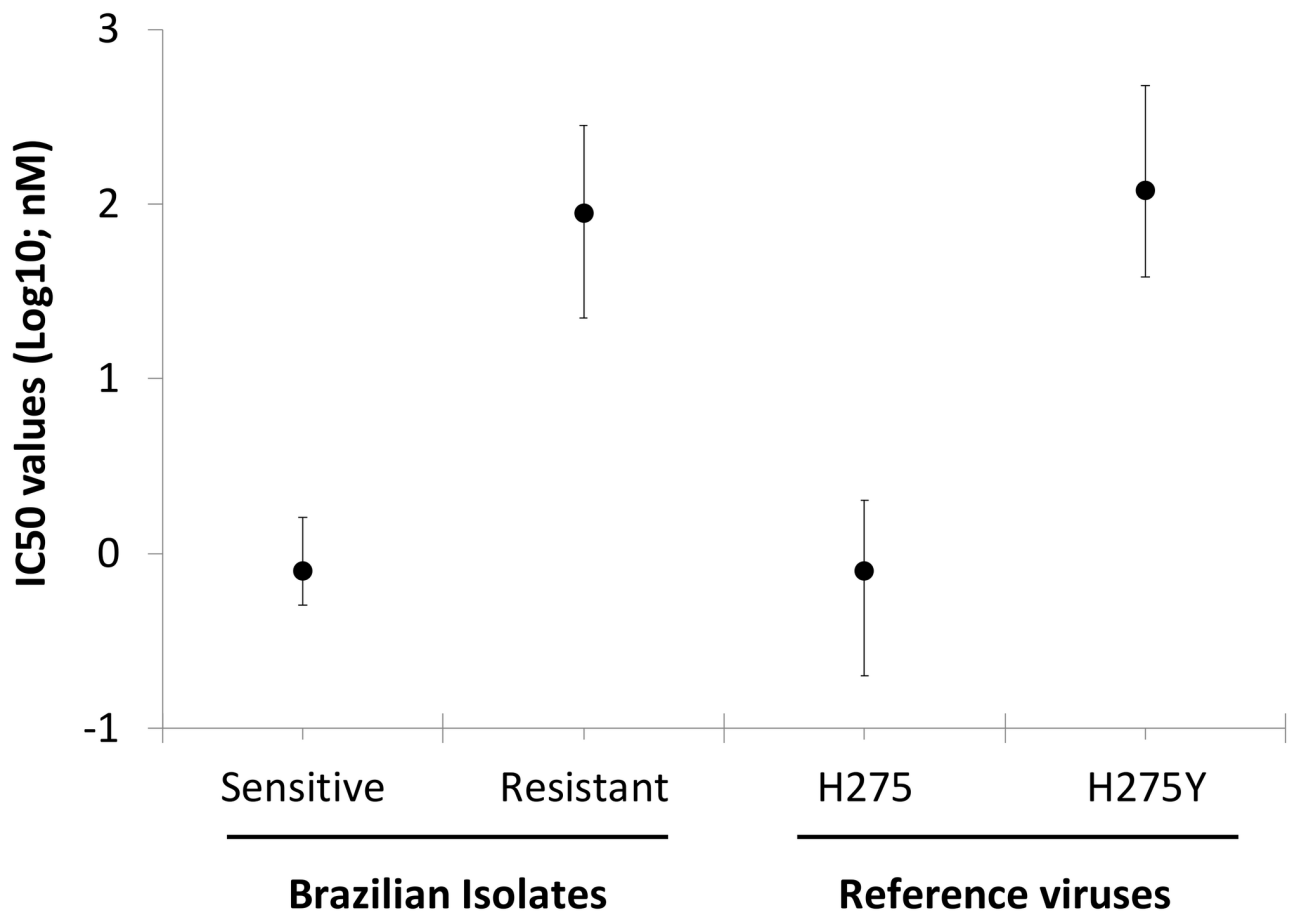
IC_50_ values for Brazilian isolates. A total of 56 A(H1N1)pdm09 isolates (at maximum three passages in MDCKs) were titred, and their IC_50_ values were determined using NA-Star^TM^ assay (Brazilian). For comparison, the WT (H275) and resistant (H275Y) strains of A(H1N1)pdm09 virus A/Perth/265/2009 and A/Perth/261/2009, respectively. The data represent the mean ± maximum and minimum ranges.

As we mentioned above, our sampling was orientated by the screening of H275Y and S247N mutations by pyrosequencing. Among these successfully analysed samples, Sanger sequencing was performed with a representative number of specimens, 56 (GenBank accession numbers: KC984872-KC984935). This subset of specimens had WT amino acid residues at positions V116, I117, E119, Q136, K150, D151, D199 and I223. Seven mutant viruses were detected, by both methodologies, 2 S247N and 5 H275Y viruses (Table 1). Functional assays were successfully performed with 56 virus isolates. The IC_50_ values for sensitive and resistant viruses were 0.8 ± 0.7 nM and 89 ± 23 nM (mean ± SD), respectively (Figure 1). 

**Table 1:** Clinical and virological aspects of patients in which OST-resistant strains of influenza were detected.

**Patient**	**Mutations**	**Region**	**State**	**Age ^a^**	**Gender**	**Symptoms date**	**Collection date**	**Oseltamivir (Beginning date)**	**Deceased**	**Oseltamivir before sampling**
**1**	H275Y	Southern	RS	26	M	24-Aug-09	25-Aug-09	NI	NI	NI
**2**	S247N	Southern	RS	3^*^	M	NI	2-Jul-11	NI	Yes	NI
**3**	H275Y	Southern	SC	36	M	14-May-12	20-May-12	21-Jul-12	No	No
**4**	H275Y	Southern	PR	12	M	21-Jun-12	22-Jun-12	22-Jul-12	No	No
**5**	H275Y	Southern	RS	34	M	28-Jun-12	2-Jul-12	1-Jul-12	No	Yes
**6**	H275Y	Southern	RS	28	M	22-Jun-12	27-Jun-12	25-Jun-12	No	Yes
**7**	S247N	Southeast	MG	37	F	1-Jul-12	5-Jul-12	5-Jul-12	Yes	NI

^a^ Years old; * months old; NI – not informed

The S247N mutation was identified in viruses from 2 patients (Table 1), including a 37-year-old female from Minas Gerais (MG) with SARI and diffuse lung infiltrates who was admitted to the intensive care unit (ICU). She had OST therapy initiated 4 days after the onset of illness, and her NPA sample was collected before such treatment. She passed away as a complication of influenza infection. The other patient was a three-month-old male with Down syndrome and pulmonary complications, from the state of RS. He required hospitalisation and died due to influenza infection. No information about OST use was recorded. 

The H275Y A(H1N1)pdm09 virus was found in four other patients (Table 1), from the Santa Catarina (SC), Parana (PR) and RS states. The patient from SC, a 36-year-old male, required ICU admission but had a benign clinical outcome. Importantly, OST was administered to him 7 days after the onset of illness, and NPA was collected before that. The patient from PR, a 12-year-old male, rapidly received OST treatment, which had been initiated within 24 h after the beginning of the symptoms. His sample was collected before treatment. He had a benign clinical outcome. The patients from RS were 28- and 34-year-old males displaying pneumonia. Both patients survived. These two patients received OST before sample collection, and 100 % of H275Y quasi-species were observed in samples from these individuals, both by pyrosequencing and Sanger sequencing (Table 1). This rapid emergence is very unusual, suggesting that they might have been primarily infected by the resistant virus. Of note, all these seven individuals had not been vaccinated against influenza. 

Considering that the state of RS was more severely affected during the pandemic [19] and has a temperate climate, we also studied an additional 100 samples from this state from 2009 to further evaluate the previous existence of H275Y virus in Brazil. We indeed found one sample from an immunocompromised 28-years-old male with the H275Y mutation. Information on the use of OST or clinical outcome was not available for this patient. In fact, he passed away a month after the onset of illness, and reasons other than influenza cannot be ruled out. 

## Discussion

In this work, some of the OST-resistant viruses and viruses with decreased susceptibility to this drug were detected after the beginning of antiviral treatment, such as for the two patients from RS and for another from MG (Table 1, patients 5 to 7). Although these results may suggest the emergence of mutant virus due to selective pressures imposed by the treatment, the absence of any sample prior to treatment makes it difficult to confirm. Moreover, we detected in patients 5 to 7 100 % of quasi-species in their samples (by both pyrosequencing and Sanger) at zero to two days after OST initiation. This short time frame may also jeopardise the conclusion that mutant viruses were generated by selective pressure. Thus, the suggestion of community transmission of H275Y virus may be not limited to patients from PR and SC states, for whom resistant viruses were detected before antiviral therapy initiation occurred (Table 1, patients 3 and 4) and to the resistant virus found in 2009 in a patient with scarce medical records. Thus, continuous and systematic studies on the circulation of NAI-resistant strains of influenza in Brazil should be conducted to confirm or rule out this suggestion. In fact, community transmission of H275Y viruses has been described in other parts of the world [4,5] and is possible due to a new balance, which favours viral fitness, between NA and haemagglutinin (HA) activities in this strain [20].

Moreover, considering the continental-wide distribution of Brazil from the equatorial line to sub-tropical areas, the seasonality and impact of respiratory viruses may be greatly variable throughout Brazilian territory. The southern states of Brazil, RS, SC and PR, generally have a temperate climate and more marked seasonality of influenza than other states. As a result of that, during the pandemic for example, the mortality ratio of A(H1N1)pdm09-confirmed deaths for Brazil as a whole was 1.1 % (1.1 case per 100,000 inhabitants), whereas the ratio for the southern region was 3.0 % [19]. Thus, high influenza activities in these states could lead to more rapid viral evolution, which in the presence of antiviral drugs would lead to the selection of resistant strains that may be endowed with the ability for sustained transmission [20]. Considering samples analysed in this study and in our previous publication [7], over 913 samples of 6303 A(H1N1)pdm09-confirmed cases (> 95 % confidence interval; CI) were interrogated for the presence of H275Y mutation from 2009 to 2012 (Table 2). This is the largest laboratory-based influenza-related study performed in Brazil in terms of number of cases investigated. We found 4 out of 5 OST-resistant H275Y in 2012, when nearly all A(H1N1)pdm09-confirmed cases (> 97 %) were studied for rresistance emergence (Table 2). In 2012, an incidence of approximately 1.19 % of OST-resistant strains can be estimated in Brazil, which is similar to what have been found in others parts of the world [4,5]. Of note, our study was carried out with convenience samples received by the National Influenza Center, rather than being a classical epidemiological designed investigation. 

**Table 2:** Summary of samples tested for the detection of H275Y mutation in A(H1N1)pdm09 viruses from 2009 to 2012.

			**% Confidence Intervals**				
**Years**	**Suspected cases sent to NIC-Brazil**	**A(H1N1)pdm09-confirmed cases**	**95**	**90**	**99**	**Studied cases for A(H1N1**)**pdm09-carrying H275Y**	**A(H1N1)pdm09-carrying H275Y**
2009	16936	5655	360	259	594	315*	1
2010	1789	140	103	93	116	100*	0
2011	1431	163	114	102	130	163	0
2012	2016	345	183	152	228	335	4
**Total**	**22172**	**6303**	**760**	**606**	**1068**	**913**	**5**

* The numbers represent the sum of the cases presented in this study and in our previous publication [7].

Another highlight from our study would be the detection of OST-resistant viruses in Foz do Iguaçu city (Iguaçu Falls) in the state of PR. This city forms a tri-border with the Argentine and Paraguayan cities of Puerto Iguazu and Ciudad de Leste, respectively. Foz do Iguaçu is a cosmopolitan city with over 200,000 inhabitants and more than 1,700,000 inbound and outbound flights and buses annually at the city’s international airport and bus station [21]. The flow of people between Foz do Iguaçu and Ciudad de Leste is estimated to be over 16,500 persons daily [21]. Therefore, it seems plausible to believe that OST-resistant A(H1N1)pdm09 could also be found in Paraguay. To our knowledge, there are no scientific studies on influenza resistance to NAIs in Paraguay. In Argentina, few cases of OST-resistant strains of pandemic virus have been described in its capital [22]. No information about H275Y virus strains in the northeastern region of Argentina was found.

Therefore, our data reinforce the importance of enhanced surveillance on the emergence of NAI-resistant strains of influenza in developing countries because such emergence may drive critical decisions in public health, ranging from drug stockpiling to health interventions.
